# New Perspectives on Antimicrobial Agents: Pivmecillinam

**DOI:** 10.1128/aac.01824-24

**Published:** 2025-07-21

**Authors:** Brandon K. Hawkins, Helen Ding, Melphine M. Harriott, James L. Wang

**Affiliations:** 1 Department of Clinical Pharmacy and Translational Science, University of Tennessee Health Science Center College of Pharmacy, Knoxville, Tennessee, USA; 2 Department of Pharmacy, University of Tennessee Medical Center 21823, Knoxville, Tennessee, USA; 3 Healthcare-Associated Infections and Antimicrobial Resistance Program of the Communicable and Environmental Diseases and Emergency Preparedness Division, Tennessee Department of Health 5719, Nashville, Tennessee, USA; 4 Division of Infectious Diseases, University of Tennessee Medical Center 21823, Knoxville, Tennessee, USA; 5 Department of Internal Medicine, Graduate School of Medicine, University of Tennessee Health Science Center, Knoxville, Tennessee, USA; University of Pittsburgh School of Medicine, Pittsburgh, Pennsylvania, USA

**Keywords:** pivmecillinam, Enterobacterales, clinical data, review, urinary tract infections, extended-spectrum beta-lactamase producers, amdinocillin

## Abstract

Pivmecillinam is a prodrug of mecillinam, a semi-synthetic penicillin, that has recently been U.S. Food and Drug Administration approved for the treatment of uncomplicated urinary tract infections (UTIs) in the United States. Mecillinam demonstrates activity against a variety of common uropathogens, including Enterobacterales such as *Escherichia coli* and *Klebsiella pneumoniae*. Despite being available internationally for more than four decades, mecillinam retains high susceptibility rates against a variety of Enterobacterales, including some that produce extended-spectrum-β-lactamases (ESBL) and carbapenemases. Given extensive clinical experience and history with this agent outside the United States, this review highlights the available literature on pivmecillinam regarding susceptibility testing and implementation, empiric antibacterial activity with a focus on ESBL-producing isolates, optimal treatment dose, and duration, as well as adverse effects and tolerability. In the United States, the current lack of approved susceptibility tests will limit pivmecillinam use to empiric therapy. There is significant heterogeneity among clinical studies, limiting the ability to identify an optimal treatment regimen for most pathogens with certainty. Comparative data regarding pivmecillinam against other agents used for UTI treatment are similarly limited but do not suggest it is significantly different in terms of clinical outcomes when considering specific treatment regimens. Clinical data regarding pivmecillinam’s use in UTI due to ESBL-producing Enterobacterales are less robust but appear supportive if used at higher doses and for longer durations. Pivmecillinam is generally well tolerated, with gastrointestinal-related complaints being the most common. In the future, the availability of susceptibility testing and clinical data for pivmecillinam against OXA-48-like-producing Enterobacterales could expand its therapeutic utility for UTI treatment.

## PERSPECTIVE

Urinary tract infections (UTIs) represent a significant clinical challenge in the United States (U.S.), compounded by a rise in antimicrobial resistance (1). Specifically, the increasing prevalence of extended-spectrum β-lactamase-producing Enterobacterales (ESBL-E) has limited effective oral treatment options (1, 2). Given the need for alternative antimicrobial agents, the recent U.S. Food and Drug Administration (FDA) approval of pivmecillinam for uncomplicated UTI (uUTI) in female patients aged 18 and older caused by *Escherichia coli*, *Proteus mirabilis*, and *Staphylococcus saprophyticus* offers a promising development.

Pivmecillinam is the prodrug of mecillinam, a synthetic penicillin derivative with a long-established history of use in Europe and Canada that has demonstrated remarkably low resistance rates despite decades of clinical application (3, 4). Given that the clinical history of mecillinam is extensive and spans several decades, a comprehensive review of all available literature is beyond the scope of this manuscript. Early publications in English with less specific study designs, limited data, and outcome measures are included, though the ability to draw firm conclusions from them regarding pivmecillinam’s contemporary use in the U.S. is limited.

## OVERVIEW

Initial pharmacokinetic studies suggested that oral bioavailability of mecillinam was poor, although this was improved when administered as a pivaloyl-oxymethyl ester prodrug (i.e., pivmecillinam) (5). Despite the prodrug formulation, oral bioavailability is estimated to be 25–35% and achieves high concentrations in the kidneys and urine (6). Approximately 80% of the absorbed dose is excreted as active drug (7), with urine concentrations achieving >200 mg/L within the first 6 h following a 200 mg dose (8). Higher doses (e.g., 400 mg) have been observed to produce peak urine concentrations around 300 mg/L, with mean urine concentrations of 8 mg/L 12 h after the dose (9). The optimal fraction of time above minimum inhibitory concentration (fT > MIC) for stasis has been reported to be 30–35% (9). The fT > MIC for 1−log_10_ reduction is unknown.

Like other β-lactams, pivmecillinam exerts its antimicrobial effect by binding to penicillin-binding proteins (PBP), with particularly high binding specificity for PBP-2 (10). This contrasts with many other β-lactams that preferentially bind PBP-1A, -1B, and/or -3. *In vitro* activity against common uropathogens (e.g., *E. coli* and *Klebsiella pneumoniae*), including ESBL-, AmpC-, and certain carbapenemase-producing isolates, appears excellent (7, 11–14). Other common urinary pathogens, such as *S. saprophyticus*, may test resistant *in vitro*, but multiple studies have demonstrated *in vivo* success (15).

From a safety perspective, carnitine depletion is the primary concern associated with pivmecillinam use. The pivalic acid moiety liberated from pivmecillinam following ingestion is eliminated via urinary excretion after conjugation with carnitine (16). This adverse effect, more pronounced with prolonged durations of therapy (22–30 months) but still observable with short-term use, can persist for up to 10 days post-cessation (16). Consequently, pivmecillinam is contraindicated in patients with carnitine deficiency and is not recommended with concurrent use of valproic acid or other pivalate-generating drugs. Additionally, due to the risk of acute porphyria, pivmecillinam is contraindicated in individuals with this condition (17). The risk of *Clostridioides difficile* infection (CDI) associated with pivmecillinam is considered low, with an *in vitro* human gut model demonstrating no germination, proliferation, or high-level toxin production in the presence of mecillinam (18). Drug interactions primarily involve agents that impact the metabolism or are substrates of organic anion transporters 1 and 3 (e.g., methotrexate), as pivmecillinam itself is a substrate of these proteins (7).

Given that pregnant women are disproportionately impacted by asymptomatic bacteriuria and UTI, pivmecillinam’s safety in this population has been extensively evaluated. Early studies of fetal impacts focused on the potential effects of carnitine depletion, such as congenital abnormalities, pre-term delivery, low birthweights, low Apgar scores, and hypoglycemic episodes. After adjusting for maternal age and smoking, a registry study found no significant differences in these outcomes between fetuses with and without exposure to pivmecillinam, though the authors noted the associated confidence intervals were relatively imprecise (19). A larger study confirmed these results with greater certainty (20).

Pivmecillinam’s association with congenital malformations was further evaluated in two Danish registry studies. While the initial study focused on orofacial clefts noted a significant increase in the risk of cleft palate among pregnancies exposed to pivmecillinam in the third trimester, the authors suggested these findings could be due to confounding by indication (21). This is particularly relevant given that previous studies had identified UTI during pregnancy to be associated with an increased risk of orofacial clefts (21). This consideration was further supported by findings from a larger registry study where rates of any major congenital malformation were not elevated when comparing pivmecillinam-exposed and penicillin-exposed cohorts (aOR: 1.02; CI: 0.94–1.1) (22). The authors contend the modest risk increase observed among the pivmecillinam-exposed cohort (aOR: 1.13; CI: 1.06–1.19) as compared to those without any antibiotic exposure likely represents confounding by indication (22). This bias also appears relevant regarding other potential associations, such as pivmecillinam exposure *in utero* and the increased risk of developing childhood epilepsy (23). In this case, the authors also concluded that confounding by indication was likely implicated, as none of the associated antibiotics were known neurotoxins (23). As a whole, a detailed review of the data supports the safety of pivmecillinam use during pregnancy.

## *IN VITRO* SUSCEPTIBILITY TESTING AND IMPLEMENTATION

Although the U.S. FDA has recently approved the use of pivmecillinam, the Clinical Laboratory Standards Institute (CLSI) M100 initially published MIC and zone diameter breakpoints, as well as interpretations for antimicrobial susceptibility testing, in 2002 (24). As pivmecillinam is a prodrug, mecillinam is used for *in vitro* susceptibility testing. The European Committee on Antimicrobial Susceptibility Testing (EUCAST) v15.0 has also established breakpoints for mecillinam (25), while the FDA recognizes the most recent CLSI breakpoints, M100 Ed35 (Table 1). The quality control ranges for disk diffusion and MIC testing are consistent between CLSI M100 Ed35 and EUCAST v15.0 (26). The CLSI mecillinam breakpoints apply exclusively to *E. coli*, whereas the EUCAST breakpoints apply to *E. coli* and several other Enterobacterales, including *Citrobacter* spp., *Enterobacter* spp., *Klebsiella* spp., *P. mirabilis*, and *Raoultella* spp. CLSI M100 Ed35 and EUCAST v15.0 breakpoints specifically pertain to urine specimens, with EUCAST v15.0 further specifying that these breakpoints are relevant only for uUTI. CLSI M100 Ed35 does not specify the dosage corresponding to the published breakpoints, while EUCAST v15.0 bases its breakpoints on an oral dosage regimen of 200–400 mg three times daily.

**TABLE 1:** Mecillinam *in vitro* antimicrobial susceptibility testing breakpoints^*b*^

Standards	Breakpoints						Approved organisms	Specimen type
	MIC (µg/mL or mg/L)^*a*^			Zone diameter (mm) (10 µg disk)				
	S	I	R	S	I	R		
CLSI M100-Ed35 (2025)	≤8	16	≥32	≥15	12–14	≤11	*E. coli*	Urine
EUCAST 2025 v15.0	≤8	-	>8	≥15	-	<15	*Citrobacter* spp. *E. coli* *Enterobacter* spp. *Klebsiella* spp. *P. mirabilis* *Raoultella* spp.	Urine (uncomplicated UTI only)
FDA	CLSI M100 standard recognized							

^*a*^ CLSI reports MIC and zone diameters in micrograms per milliliter (µg/mL), while EUCAST provides these measurements in milligrams per liter (mg/L); Abbreviations: CLSI, Clinical Laboratory Standards Institute; EUCAST, European Committee on Antimicrobial Susceptibility Testing; FDA, U.S. Food and Drug Administration; MIC, minimum inhibitory concentration; mm, millimeters; UTI, urinary tract infection.

^*b*^ - indicates that there is no MIC or Zone diameter for mecillinam at the specified breakpoint for these organisms.

CLSI M100 Ed35 and EUCAST v15.0 designate agar dilution as the reference method for assessing mecillinam MIC. This preference may arise from the trailing effects observed when mecillinam is tested using broth microdilution (27). Furthermore, media composition significantly influences mecillinam MIC determination in broth media. A previous study demonstrated that MIC testing conducted in Muller-Hinton broth may yield inconsistent results for mecillinam (10). Both osmolality and ion concentration appear to affect the MIC, with increases in these parameters resulting in elevated MICs (10).

Although mecillinam gradient diffusion strips are commercially available, their primary use remains within research environments rather than clinical laboratory settings (28–31). Studies comparing gradient diffusion and automated MIC methods to agar dilution have shown that these alternative methodologies demonstrate lower accuracy testing than agar dilution (32, 33). A recent investigation assessed mecillinam susceptibility testing for *C. koseri, E. cloacae*, *E. coli*, and *Klebsiella* spp. using three methodologies: VITEK 2 (automated system), ETEST (gradient diffusion), and the reference agar dilution method (33). International Organization for Standardization guidelines were used to evaluate essential agreement (EA) and categorical agreement (CA) among the methods employed. Among the tested organisms, only *E. coli* and *C. koseri* met the acceptable threshold of ≥90% for EA, defined as ±1 twofold dilution of the MIC reference method when using both VITEK 2 and ETEST methodologies. However, these organisms also exhibited higher rates of very major errors, significantly exceeding the acceptable rate of ≤3% for both methods. This finding aligns with other studies comparing gradient diffusion with agar dilution for Enterobacterales with very major errors of 12.2% (32). Regardless of the methodology, the comparison study indicated that *P. mirabilis* consistently failed to yield reproducible susceptibility results. Overall, disk diffusion was found to be more accurate than VITEK 2 or ETEST for mecillinam susceptibility testing. These findings correspond with previous studies demonstrating an excellent correlation between mecillinam disk diffusion and agar dilution (13, 34). Other automated testing platforms, such as the DxM MicroScan WalkAway Plus System, have also been used for mecillinam MIC testing and have demonstrated a favorable correlation with disk diffusion (13, 34). However, there has not been a comparison of MicroScan with the agar dilution reference method; consequently, the accuracy of these automated results remains uncertain.

In the U.S., implementing mecillinam susceptibility testing is significantly constrained by the absence of FDA-approved automated susceptibility testing platforms, disks, or gradient diffusion strips for *in vitro* diagnostics (35). Moreover, the reference method, agar dilution, is labor-intensive and impractical for small test volumes, thereby posing substantial challenges for most clinical laboratories seeking to incorporate it into their routine workflows. Disk diffusion represents a more user-friendly approach for laboratories aiming to conduct mecillinam susceptibility testing in the future. However, several factors must be considered during interpretation. The EUCAST v15.0 guidelines specify to disregard colonies within the zone of inhibition, a common finding with mecillinam disk diffusion (25). Additionally, increased discrepancies and technical uncertainties have been observed concerning zone sizes ranging from 14 to 16 mm (13), particularly for carbapenem-resistant Enterobacterales (CRE) (13). This variability may contribute to the CLSI M100 Ed35 interpretation, which defines zone diameters between 12 and 14 mm as intermediate (26). EUCAST v15.0 does not include an intermediate interpretation.

## *IN VITRO* ACTIVITY

*In vitro* studies assessing mecillinam have been predominantly conducted in European and Scandinavian countries, primarily focusing on urine isolates using disk diffusion or agar dilution and applying EUCAST v15.0 breakpoints. A recent study conducted in the U.S. analyzed 3,303 urine isolates of Enterobacterales using agar dilution and CLSI breakpoints (14). A comprehensive overview of the *in vitro* activity of mecillinam was provided in a recent review, with the following section summarily reviewing those findings and other highlights (4).

### E. coli

Mecillinam demonstrates substantial *in vitro* activity against *E. coli,* including ESBL-, AmpC-, and carbapenemase-producing isolates. For *E. coli* that do not produce ESBL or AmpC and are not resistant to carbapenems, most studies report susceptibility rates exceeding 95% (4). While a few studies indicate lower susceptibility rates, none report rates below 80% (4).

Mecillinam is active against *E. coli* expressing a range of ESBL enzymes, including CTX-M, TEM, SHV, VEB, and OXA (11, 36, 37). The ESBL *E. coli* susceptibility rate is documented to exceed 90%, with no studies reporting rates lower than 82.5% (4). MIC50 and MIC90 values using EUCAST v15.0 breakpoints for a variety of *E. coli* isolates are presented in Table 2. These reported MIC values are comparable when applying the *E. coli* CLSI M100 Ed35 breakpoints (14).

**TABLE 2:** MIC50/90 for *E. coli* with agar dilution testing (4)

	MIC50 range (µg/mL or mg/L)^*a*^	MIC90 range (µg/mL or mg/L)^*a*^	Overall MIC range (µg/mL or mg/L)^*a*^
*E. coli* (excluding ESBL, AmpC, CRE)	0.25–0.5	2–4	0.06 to >128
ESBL *E. coli*	1–2	4–8	0.06 to >128

^*a*^ CLSI reports MIC and zone diameters in micrograms per milliliter (µg/mL), while EUCAST provides these measurements in milligrams per liter (mg/L); Abbreviations: CRE, carbapenem resistant Enterobacterales; ESBL, extended-spectrum β-lactamase; MIC, minimum inhibitory concentration.

Mecillinam has also demonstrated activity against carbapenem-resistant and carbapenemase-producing *E. coli*. A study evaluating 1,943 carbapenem-resistant *E. coli* using disk diffusion reported an 84.2% susceptibility rate to mecillinam (13). Importantly, *in vitro* response appears related to the specific carbapenemase produced by these isolates. *E. coli* isolates producing OXA-48-like carbapenemase demonstrate a susceptibility rate of 92.6%, whereas NDM-producers display a susceptibility rate of 76.2%. Conversely, VIM-producing *E. coli* exhibits a significantly lower susceptibility rate of 17.6%, while KPC-producers are largely resistant (0% susceptible) (13).

### Other Enterobacterales

Multiple studies have examined the susceptibility of various Enterobacterales to mecillinam. While most isolates demonstrate susceptibility, this can vary depending on the testing methodology and breakpoints employed. In cases where established mecillinam breakpoints are lacking, most studies substitute those used for *E. coli* to interpret the data. Non-multidrug-resistant *C. freundii* and *E. cloacae* show susceptibility rates above 95% when using agar dilution and CLSI *E. coli* breakpoints, but approximately 88% when using disk diffusion and EUCAST breakpoints (14, 38). The susceptibility of *Klebsiella* varies among species, with rates ranging from 81.8% to 93.5% (14, 39, 40). ESBL-producing *Klebsiella* isolates may also demonstrate lower susceptibility compared to non-ESBL-producers (14, 38–40). *P. mirabilis* exhibits variable susceptibility, possibly due to discrepancies in testing methodologies, with rates ranging between 73.2% and 95.8% (14, 38–40). Mecillinam appears to have limited effectiveness against *Morganella morganii, Proteus vulgaris*, and *Serratia marcescens* (38, 39). Interestingly, mecillinam is active against *Salmonella* spp. and carries an indication for treatment in Europe (41, 42). Mecillinam’s efficacy against CRE or ESBL-E is similar to that observed with *E. coli*; it is active against IMI and OXA-48-like β-lactamases, less so against NDM, and with little to no activity against VIM and KPC producers (13). While *in vitro* studies have shown promising results for mecillinam against Enterobacterales, the relationship between these findings and *in vivo* responses remains ambiguous, especially for ESBL-producers (9, 43–45).

### Non-Enterobacterales

Mecillinam is ineffective against *Pseudomonas* species, *Neisseria gonorrhoeae* and gram-positive cocci, including *Enterococcus faecalis* and *S. aureus* (41, 46, 47). While *S. saprophyticus* demonstrates *in vitro* resistance to mecillinam, some reports indicate it is clinically effective in treating UTI due to this organism (15, 40, 48). Mecillinam’s high urinary concentrations may be responsible for the discordance between *in vitro* susceptibility and observed clinical cure (15).

## MECHANISMS OF RESISTANCE

Despite decades of pivmecillinam utilization in Scandinavia and throughout Europe, the *in vitro* susceptibility of Enterobacterales to mecillinam has remained stable in these regions (4, 40, 49, 50). In the U.S., mecillinam resistance among Enterobacterales remains low, with susceptibility rates fluctuating from 94.3% to 95.3% and minimal variation from 2017 to 2020 (14).

The precise mechanisms underlying mecillinam resistance remain largely unresolved, and there is currently limited knowledge regarding the mecillinam resistome. Despite this, cysteine biosynthesis modulation has been implicated as a potential mechanism (51). Resistance in clinical *E. coli* isolates has been most commonly associated with modification of the *cysB* gene, which regulates cysteine biosynthesis (29). Mutations in *cysB* upregulate genes encoding proteins involved in peptidoglycan synthesis, including PBP1B, LpoB, and FtsZ (51). Significantly, mecillinam resistance in *cysB* mutants can be mitigated by adding cysteine to the culture media (29). Moreover, *cysB* mutants demonstrate increased susceptibility to mecillinam when cultured in urine as opposed to Muller Hinton broth (30). Research indicates that *cysB* mutants are more susceptible to mecillinam in urine with lower, as opposed to higher, osmolality (30). These findings highlight potential factors, including media composition, that may influence the accuracy and reliability of mecillinam susceptibility testing within the clinical laboratory.

A 2018 study examined 2,547 urine *E. coli* isolates collected between 2014 and 2016, demonstrating complete susceptibility to β-lactam antibiotics other than mecillinam (52). Enzymatic mechanisms associated with mecillinam resistance in these isolates included penicillinase overproduction (64.4%), TEM-type or OXA-1 β-lactamase production (16.3%), ESBL production (6.7%), and chromosomal AmpC β-lactamase (2.9%) overproduction. A small percentage of isolates (9.6%) lacked a discernible mechanism. These isolates underwent further characterization, revealing a small colony morphology on Muller Hinton agar, a rounded appearance microscopically, and the presence of mutations in *cysB*. It remains unclear how disruptions in cysteine biosynthesis contribute to mecillinam resistance, but this may be attributed, in part, to elevated levels of intracellular guanosine pentaphosphate/tetraphosphate (ppGpp). These molecules serve as stress alarmones that play a crucial role in the bacterial stress response and development of antibiotic tolerance and persistence (29, 53).

Mecillinam, like other β-lactam agents, is affected by the inoculum effect, particularly in organisms that produce ESBL and AmpC enzymes (11, 12, 54). The use of a β-lactamase inhibitor can help mitigate this, with synergy observed when combining mecillinam with clavulanic acid against *E. coli* and other Enterobacterales. Reductions in MICs have also been demonstrated when mecillinam is combined with avibactam or ceftazidime-avibactam, including among ESBL- and carbapenemase-producing *E. coli* (11, 12, 54–57).

There is a report of emergent mecillinam resistance in *E. coli* following mecillinam treatment. When compared with the initial susceptible isolate, the resistant isolate exhibited a mutation in the *bla*_CTX-M-15_ gene, resulting in the alteration of *bla*_CTX-M-127_ (58). Other CTX-M derivatives, such as *bla*_CTX-M-215_, have also been found to confer high-level mecillinam resistance in *E. coli*. Interestingly, both studies utilized gradient diffusion to evaluate mecillinam susceptibility.

Data regarding β-lactam cross-resistance among bacterial isolates resistant to mecillinam are currently limited. Existing knowledge suggests minimal cross-resistance between mecillinam and other β-lactam antibiotics (59). However, *E. coli* isolates resistant to mecillinam have been observed to demonstrate resistance to amoxicillin and amoxicillin-clavulanic acid (60). Nonetheless, the specific mechanisms contributing to mecillinam resistance are not fully understood, highlighting the need for further research in this area.

## CLINICAL DATA

Assessing the efficacy of pivmecillinam relative to other agents for the treatment of UTI is difficult given the small sample sizes, varying definitions of treatment success, as well as limited pathogen reporting and statistical comparisons. Early comparison studies were also performed at a time when resistance rates among certain agents, such as fluoroquinolones, were much lower. This is further complicated by varying outcome definitions, follow-up timeframes, and agents unavailable in the U.S. (i.e., nalidixic acid and sulfamethizole). As such, there is not sufficient evidence to conclude that clinical outcomes with pivmecillinam therapy are significantly different than treatment with other agents when employing specific doses and durations of therapy. The section that follows summarizes the available English-language clinical data regarding comparative pivmecillinam dosing and duration for non-ESBL-E and ESBL-E pathogens, as well as its use in more complicated infections.

### Comparative dose and duration

Previous studies have evaluated pivmecillinam dosing strategies ranging from 200 mg two times daily to 400 mg three times daily, with durations of 3–10 days (Table 3). Given the variety of regimens reported in the literature, a 2017 meta-analysis attempted to identify an optimal regimen for the treatment of uUTI, but ultimately determined there was insufficient evidence to support an optimal dosage, frequency, and duration (61). Broadly reviewing the literature, shorter durations of therapy (i.e., 3 days) do not appear significantly less effective in terms of clinical cure than longer durations of therapy (i.e., 5–10 days) (62–65). Dosing of 200–400 mg three times per day (TID) has been reported with these short courses of therapy, without a significant difference in cure identified. However, the most robust study conducted to date by Ferry et al. did note numerically lower clinical cure among those receiving 400 mg two times per day (BID) ×3 days as compared to treatment for 7 days (55% vs 62–64%; *P* = 0.16) (66). Furthermore, patients in the 3 days group had significantly higher mean symptoms scores after completing treatment at the first follow-up period (1.33 vs 1.03 vs 0.85; *P* = 0.024). Though these differences had resolved at the final follow-up period (35–49 days), this suggests that 3 days regimens may be less effective than longer courses for clinical cure/resolution. Similar findings for early clinical cure/improvement were noted by Nicolle et al. when comparing 3 days courses of pivmecillinam and norfloxacin (82% vs 88%, *P* = 0.019) (67).

**TABLE 3:** Clinical studies comparing pivmecillinam dosing and duration^*a*^

Study	Design and population	Treatment groups for analysis	Defined outcomes	Results	Microbiology	Adverse events
Bresky (68)	Randomized, cohort study Lower UTI symptoms, age 15–30 years Follow-up: within 10 days post- treatment	PIV 200 TID ×10 days (*n* = 92) PIV 400 TID ×10 days (*n* = 78)	Bacteriologic cure: original organism eradication and negative urine culture at follow-up	Bacteriologic cure: PIV 200 TID: 89% PIV 400 TID: 90%	Disk diffusion susceptibility testing 84% Enterobacterales, of which 90% *E. coli* All Enterobacterales reported susceptible	Majority of events mild in severity GI events most common: PIV 200 TID: 11% PIV 400 TID: 19%
Marsh and Menday (62)	RCT Lower UTI symptoms, women age 15–55 years Follow-up: 4 weeks after treatment	PIV 200 TID ×3 days (*n* = 58) PIV 200 TID ×7 days (*n* = 67)	Clinical efficacy: symptom score before and after treatment Bacteriologic efficacy: not defined Symptomatic recurrence: not defined	Clinical efficacy: absolute **∆** PIV ×3 days: 4.3; ×7 days: 4.6 Bacteriologic efficacy: PIV ×3 days: 91%; ×7 days: 100% Symptomatic recurrence: PIV ×3 days: 10.3%; ×7 days: 16.4%	Disk diffusion susceptibility testing 76% Enterobacterales, of which 92% *E. coli* 95% susceptibility among Gram- Negatives	Side effects mild in severity GI events most common: PIV ×3 days: 16% PIV ×7 days: 18%
Sutlieff (63,63)	RCT Uncomplicated symptomatic UTI, age 18–55 years Follow-up: interval not reported	PIV 400 × 1, 200 TID ×3 days (*n* = 39, efficacy) (*n* = 44, safety) PIV 200 BID ×5 days (*n* = 49, efficacy) (*n* = 54, safety)	Clinical response: symptom score before and after treatment Bacteriologic cure: negative culture or new organism post-treatment	Clinical response: absolute ∆, Not statistically significant Values not reported Bacteriologic cure: PIV 200 TID ×3 days: 95% PIV 200 BID ×5 days: 96%	Disk diffusion susceptibility testing 89% Enterobacterales of which 82% *E. coli* 90% susceptibility among Gram- negatives	Side effects mild in severity GI events most common: PIV 200 TID ×3 days: 5% PIV 200 BID ×5 days: 11%
Pettersson et al. (69)	RCT Uncomplicated symptomatic UTI, women Follow-up: 3–5 days after treatment completion	PIV 200 TID ×7 days (*n* = 19, efficacy) (*n* = 27, safety) PIV 400 TID ×7 days (*n* = 22, efficacy) (*n* = 31, safety)	Cure: disappearance of bacteriuria and symptoms	Cure: not statistically significant PIV 200 TID: 84.2% PIV 400 TID: 77.3%	Susceptibility testing method not reported 71% Enterobacterales of which 90% *E. coli* 96% susceptibility among Enterobacterales	Side effects mild to moderate in severity PIV 200 TID: 7% PIV 400 TID: 23%
Richards (65,65)	RCT Uncomplicated symptomatic UTI, women age 18–55 years Follow-up: 7 days after treatment assignment	PIV 400 BID ×3 days (*n* = 91, efficacy) (*n* = 92, safety) PIV 400 BID ×7 days (*n* = 84, efficacy) (*n* = 86, safety)	Clinical efficacy: symptom score before and after treatment Bacteriologic success: original organism eradication, irrespective of new pathogen	Clinical efficacy: absolute **∆** PIV ×3 days: 6.35; ×7 days: 6.55 Symptom-free, overall: PIV ×3 days: 67%; PIV × 7 days: 62% Bacteriologic efficacy: PIV ×3 days: 95%; ×7 days: 100% 4 weeks of symptomatic recurrence, # patients: *P* = NS PIV ×3 days: 6; PIV ×7 days: 2	Disk diffusion susceptibility testing 90% Enterobacterales of which 100% *E. coli* 95% susceptibility among pre-treatment *E. coli* isolates	Side effects mild in severity GI events most common: PIV ×3 days: 5% PIV ×7 days: 10%
Skinner et al. (70)	Clinical trial Uncomplicated symptomatic UTI, women age 18–55 years Follow-up: 7 days after treatment completion	PIV 200 BID ×5 days (*n* = 183, efficacy) (*n* = 185, safety) PIV 400 BID ×5 days (*n* = 109, efficacy) (*n* = 111, safety)	Clinical success: symptom score before and after treatment Bacteriologic success: absence of original organism post-treatment	Clinical success: absolute **∆** PIV 200 BID ×5 days: 6.74 PIV 400 BID ×5 days: 6.73 Bacteriologic success: *P* < 0.05 PIV 200 BID ×5 days: 100% (38/38) PIV 400 BID ×5 days: 85% (22/26)	Disk diffusion susceptibility testing 92% Enterobacterales of which 97% *E. coli* 98% susceptibility among pre-treatment Enterobacterales	Side effects mild in severity GI events most common: PIV 200 BID ×5 days: 3% PIV 400 BID ×5 days: 10%
Gordin et al. (71)	Open-label, RCT Most (96%) with lower UTI symptoms, women age 17–63 Follow-up: First follow-up at 3–5 days post-assignment, second follow-up at 4 weeks after treatment	PIV 200 TID ×3 days (*n* = 36; 3–5 days of efficacy) (*n* = 27; 4 weeks of efficacy) PIV 200 TID ×10 days (*n* = 34; 3–5 days of efficacy) (*n* = 26; 4 weeks of efficacy)	Bacteriologic cure: <10^4^ bacteria/mL in urine sample at both follow-up periods Reinfection: significant growth of new isolate after treatment at both follow-up periods Relapse: infection caused by original bacteria after initial cure at second follow-up	Bacteriologic cure: no statistically significant difference at 3–5 days or 4 weeks 3–5 days: PIV ×3 days: 83.3%; PIV ×10 days: 79.4% 4 weeks: PIV ×3 days: 81.5%; PIV ×10 days: 92.3% Reinfection: no statistically significant difference at 3–5 days or 4 weeks 3–5 days: PIV ×3 days: 0%; PIV ×10 days:12% 4 weeks: PIV ×3 days: 4%; PIV ×10 days: 0% Relapse: no statistically significant difference at 4 weeks PIV ×3 days: 15%; PIV ×10 days: 8%	Disk diffusion susceptibility testing 85% Enterobacterales of which 87% *E. coli* 99% susceptibility among pre-treatment Enterobacterales	No side effects reported during treatment with PIV Side effects mild in severity reported after treatment: PIV ×3 days: 3% vaginal candidiasis; 3% self-limiting rash PIV ×10 days: 6% vaginal candidiasis; 3% self-limiting rash
Pitkäjärvi et al. (64)	Open-label, RCT Lower UTI symptoms, women age 16–65 years Follow-up: 5 days and 4–5 weeks after treatment completion	PIV 200 TID ×7 days (*n* = 148, efficacy) (*n* = 171, safety) PIV 400 TID ×3 days (*n* = 151, efficacy) (*n* = 174, safety)	Clinical effect: presence or absence of urinary symptoms Bacteriologic effect: not defined	Clinical effect: not statistically significant at 5 days of follow-up PIV 200 TID ×7 days: 84% PIV 400 TID ×3 days: 91% Bacteriologic effect: not statistically significant PIV 200 TID ×7 days: 91% PIV 400 TID ×3 days: 94%	Susceptibility testing method not reported 79% Enterobacterales of which 95% *E. coli* 100% susceptibility among *E. coli* isolates	Side effects mild in severity: PIV 200 TID ×7 days: 5% PIV 400 TID ×3 days: 4%
Ferry et al. (66)	Double-blind, RCT Lower UTI symptoms, women ≥ 18 years Follow-Up: days 8–10 and 35–49 post-assignment	Control (*n* = 425) Placebo (*n* = 227) Only patients with significant bacteriuria included in analysis: PIV 200 TID ×7 days (*n* = 213) PIV 200 BID ×7 days (*n* = 214) PIV 400 BID ×3 days (*n* = 216)	Clinical cure: no symptom persistence during and post-therapy Bacteriologic cure: eradication of initial bacteriuria at follow-up Adverse events: assessed at both follow-up visits	Clinical cure days 8–10; 35–49: not statistically significant PIV 200 TID ×7 days: 62%, 72% PIV 200 BID ×7 days: 64%, 65% PIV 400 BID ×3 days: 55%, 68% Bacteriologic cure days 8–10 35–49: P < 0.001 PIV 200 TID ×7 days: 93%, *P < 0.001*; 89%, *P = 0.21* PIV 200 BID ×7 days: 94%, *P < 0.001*; 83%, *P = 0.21* PIV 400 BID ×3 days: 84%, *P < 0.001*; 86%, *P = 0.21*	Susceptibility testing not performed 67% Enterobacterales, of which >90% *E. coli*	Majority of events mild to moderate in severity GI events most common: PIV 5–8%, Placebo 4%

^*a*^ BID: Two-times per day; PIV: pivmecillinam; RCT: randomized controlled trial; TID: three-time per day.

Bacteriologic cure features prominently in older studies, either alone or in combination with variations of clinical cure/improvement. Because of the heterogeneity, or in some cases the absence, of definitions used for bacteriologic cure, direct comparisons of results are difficult and often conflicting. Some studies included isolated pathogens for which pivmecillinam has no *in vitro* activity (e.g., Enterococci, *Pseudomonas* spp., and *S. saprophyticus*). The persistence of these organisms in culture following treatment is thus more likely and does not necessarily indicate pivmecillinam therapy failure. However, a recent U.S. FDA analysis suggests that clinical cure with microbiologic failure is associated with an increased risk of late clinical failure (72). In the largest study available, a significantly lower rate of bacteriologic cure was observed among the 400 mg TID ×3 days group relative to the 7 days regimens (84% vs 93–94%, *P* < 0.001), but symptom recurrence was similar at the late follow-up period (35–49 days) for all pivmecillinam regimens (12–13%) (66).

Considering the limitations of the available literature, it does not appear that longer durations of pivmecillinam therapy are significantly more effective in terms of clinical cure than 3 days treatment courses. However, shorter courses (i.e., 3 days) appear to be associated with reduced, but persistent, urinary symptoms shortly after treatment cessation as compared to 7 days regimens. This was especially pronounced in the treatment of UTI due to *S. saprophyticus,* where Pitkajarvi et al. found clinical resolution was significantly lower in the 3 days group (71% vs 94%, *P* = 0.017). From the limited data available, higher doses also do not appear to confer any clinical benefit, though they may contribute to higher rates of GI side effects (69, 70). Ferry et al. concluded that pivmecillinam 200 mg BID ×7 days was likely the preferred regimen as outcomes were comparable to the 200 mg TID ×7 dose, but had higher cure rates relative to the 3 days regimen with lower pill burden (66). Notably, the FDA-approved pivmecillinam dose is 185 mg (200 mg pivmecillinam hydrochloride) TID for 3–7 days (7).

### Dose, duration, and outcomes associated with ESBL producers

While literature supports the use of pivmecillinam against several Enterobacterales, including those that produce β-lactamases, clinical data concerning the efficacy of pivmecillinam against specific mechanisms of resistance are limited despite observed *in vitro* activity. Elevated pivmecillinam MICs and an inoculum effect have been reported in ESBL-producing organisms; however, high urinary and kidney concentrations may mitigate this effect and preserve *in vivo* efficacy for UTI (8, 12).

The earliest studies that explored clinical outcomes of pivmecillinam for ESBL-E UTI involved small sample sizes. One study included eight women with uUTI caused primarily by ESBL-producing *E. coli.* Investigators observed high clinical but low bacteriological cure rates, with most patients receiving pivmecillinam 200 mg twice daily ×7 days (73). Another study included patients with cystitis receiving pivmecillinam doses of 200–400 mg TID, again primarily caused by ESBL-producing *E. coli,* with the majority treated with a longer 5–7 days of course. While follow-up was only available for 19 patients, 84% experienced clinical success, and only 22% experienced relapse (74). Despite being limited by small sample sizes, these initial findings supported the clinical efficacy of pivmecillinam against broad-spectrum β-lactamase-producing isolates.

To better investigate the role of pivmecillinam in ESBL-E infections in a larger population, Søraas et al. conducted a population-based study comparing agents for community-acquired UTI caused by ESBL-producing versus non-ESBL-producing *E. coli*, with pivmecillinam 200 mg TID ×7 days or longer being the most common dosing regimen. Treatment failure was higher in the ESBL group, which may be explained by increased prior overall exposure to antimicrobials, as evidenced by significantly higher resistance rates in this group for all agents other than nitrofurantoin. Resistance prevalence among ESBL-producing *E. coli* was significantly higher for mecillinam (0.4% vs 6.2%; *P* = 0.001), ciprofloxacin (7.7% vs 53%; *P* < 0.001), and trimethoprim-sulfamethoxazole (27% vs 72%; *P* < 0.001). Treatment failure remained higher in the ESBL cohort among those who received mecillinam (44% vs 14%), but the rate was lower than in the overall ESBL cohort (53% vs 44%), suggesting that mecillinam retains clinical value relative to other treatment options despite reduced susceptibility against ESBL-E (75).

Subsequently, a prospective observational study specifically investigated pivmecillinam for uUTI caused by ESBL-producing versus non-ESBL-producing *E. coli* (76). Patients received either 200 mg or 400 mg TID, with over half of the ESBL cohort receiving the higher dose. It is important to note that while treatment failure was higher in cases caused by ESBL-producing *E. coli*, this effect was not observed in patients receiving 400 mg TID, regardless of duration. This higher dose may be more likely to achieve a greater fT > MIC in pathogens with elevated MICs, a critical parameter for β-lactam activity. Use of the lower 200 mg dose for ≤5 days was associated with an increased risk of treatment failure when treating ESBL-producing *E. coli* UTI. *In vitro* resistance to pivmecillinam was comparable between ESBL-producing *E. coli* and non-ESBL-producing *E. coli*, an observation also true for nitrofurantoin but not for trimethoprim/sulfamethoxazole or ciprofloxacin. These observations indicate that pivmecillinam dose optimization may be relevant to improving clinical efficacy for UTI caused by ESBL-E (76).

Another large population-based study compared empiric agents for UTI caused by ESBL-producing and non-ESBL-producing *E. coli*, a substantial number of which were likely complicated. Specific doses and durations were not described, but increased treatment failure was again observed in the ESBL group, a trend consistent for each individual agent other than nitrofurantoin. Similarly to findings from previous literature, although pivmecillinam showed higher treatment failure rates in the ESBL cohort, this was lower than for other non-nitrofurantoin agents. Additionally, pivmecillinam and nitrofurantoin resistance rates were again found to be low in both groups but relatively higher for ESBL-producing isolates (Pivmecillinam: 2.5% vs 10.7%; Nitrofurantoin: 0.8% vs 2.9%) (77).

Most recently, a prospective multicenter observational study investigated clinical cure rates at 10–14 days post-treatment and 3 months relapse for patients with UTI caused by ESBL-E, predominantly *E. coli* (44). Two groups, febrile UTI and lower UTI, were assessed; however, only a small number of patients with febrile UTI received pivmecillinam. Among patients with lower UTI, most received either pivmecillinam 200 mg TID or nitrofurantoin. The overall cure rate was approximately 80% but lower for those receiving ≤5 days of treatment, although this finding was not statistically significant. Both nitrofurantoin and mecillinam demonstrated high susceptibility rates, 94% and 95% respectively, compared to ciprofloxacin (59%) and trimethoprim/sulfamethoxazole (63%) (44).

In summary, pivmecillinam is a promising option for the treatment of ESBL-E UTI given its favorable *in vitro* activity against both ESBL-producing and non-ESBL-producing isolates. While some data suggests that fluoroquinolones may be preferred in confirmed susceptible cases, pivmecillinam’s lower resistance rates make it a more reliable empiric choice in settings where resistance rates to fluoroquinolones and other agents, such as trimethoprim/sulfamethoxazole, are known to be elevated.

### Complicated infections

Both Danish and Norwegian guidelines recommend pivmecillinam as a treatment for acute uncomplicated pyelonephritis, with studies demonstrating favorable clinical success employing intravenous (IV) mecillinam and oral pivmecillinam alone, or in combination with another β-lactam (78). Bacteriologic success, however, may be relatively lower. In studies of bacteremia secondary to a urinary source, treatment with IV mecillinam and high-dose oral pivmecillinam (400 mg QID) has yielded acceptable clinical outcomes when given as targeted therapy following an IV lead-in with standard of care agents (79, 80). Overall, evidence supporting pivmecillinam’s use in more complicated infections is relatively limited, but utilization of IV mecillinam could help overcome potential bioavailability-related concerns. Additional research and randomized controlled trials are needed before definitive recommendations can be made for routine use beyond uncomplicated UTI. At present, IV mecillinam is not available in the U.S.

## EXPERT OPINION ON ROLE IN THERAPY

Pivmecillinam’s most attractive features for use in the treatment of UTI are its durable susceptibility and low rate of CDI (4, 14, 18). Use as empiric therapy in the U.S. appears to be reasonable among isolates with high rates of susceptibility (i.e., *E. coli*, *Klebsiella* spp., *C. freundii*, and *E. cloacae* complexes), though susceptibility testing should be performed among isolates with reportedly low or variable rates of susceptibility before use. However, CLSI M100 Ed35 breakpoint limitations (i.e., MIC interpretations available for *E. coli* only) and test availability make routine susceptibility testing commercially unfeasible. *S. saprophyticus* UTI treatment with pivmecillinam can be considered, though lower rates of clinical cure and higher rates of bacterial persistence should be expected (15, 71).

The variety of study designs, sizes, and outcomes employed in the reviewed literature makes it difficult to confidently determine the most optimal pivmecillinam regimen. The EMA recommends a regimen with a 400 mg loading dose, followed by 200 mg TID for 3 days in total, while the FDA recommends 200 mg TID for 3–7 days (7, 42). With no standardized regimen and the inability of a meta-analysis to identify significant differences among various dosing schemes and durations (61), a broad assessment of the available clinical literature seems pertinent. Considering overall clinical response and symptom resolution, 3 days of therapy appears suboptimal versus 7 days (66, 67). However, 200 mg is comparable to 400 mg for non-ESBL-E isolates (48, 66, 68–70). BID dosing is similar in efficacy to TID administration with a lower daily pill burden (48, 63, 66). Therefore, pivmecillinam 200 mg BID for 7 days seems to be the optimal empiric regimen for non-ESBL-E, with the potential benefit of reduced GI side effects associated with the 200 mg dose (68–70). However, TID dosing may provide some benefit in the treatment of ESBL-E and is likely preferred in these cases. Given signals of increased clinical failure with the lower dose (i.e., 200 mg) and shorter duration of treatment (i.e., ≤5 days), a regimen of 400 mg TID for 7 days may be reasonable when treating ESBL-E (44, 75, 76). Whether pivmecillinam could be used for the treatment of UTI due to carbapenemase-producing Enterobacterales remains unclear. Though clinical data are lacking, retained *in vitro* susceptibility for OXA-48-like carbapenemase producers appears promising (31).

## CONCLUSIONS

While pivmecillinam expands treatment options for uUTI, its oral formulation, durable susceptibility, and activity against ESBL-E underscore its unique therapeutic potential. Despite its promise, limitations and practical implementation of susceptibility testing in the U.S. may hamper its clinical utility outside of select Enterobacterales with high reported susceptibility rates. Direct comparisons to other agents used for uUTI are limited but suggest similar clinical outcomes when dose and duration are optimized. Future clinical research on its use for CRE uUTI may clarify the potential utility for these organisms.
